# Smoking Affects the Predictive Roles of Antioxidant Enzymes in the Clinical Response to Risperidone in Schizophrenia: A Large-scale Cohort Study

**DOI:** 10.2174/1570159X21666230502125800

**Published:** 2023-08-15

**Authors:** Meihong Xiu, Xiuli Song, Hanlun Yang, Xingjuan Huang, Fengchun Wu, Xiangyang Zhang

**Affiliations:** 1 Peking University HuiLongGuan Clinical Medical School, Beijing HuiLongGuan Hospital, Beijing, China;; 2 Clinical Psychology, Yantai Affiliated Hospital of Binzhou Medical University, Yantai, China;; 3 Department of Psychiatry, The Affiliated Brain Hospital of Guangzhou Medical University, Guangzhou, China;; 4 Department of Biomedical Engineering, Guangzhou Medical University, Guangzhou, China;; 5 Guangdong Engineering Technology Research Center for Translational Medicine of Mental Disorders, Guangzhou, China;; 6 CAS Key Laboratory of Mental Health, Institute of Psychology, Beijing, China

**Keywords:** Schizophrenia, tobacco smoke, risperidone, antioxidant enzyme activity, CAT activities, positive and negative syndrome scale (PANSS)

## Abstract

**Objectives:**

There is overwhelming evidence of the relationship between smoking and schizophrenia (SZ). Tobacco smoke is considered to ameliorate the symptoms and reduce the side effects of antipsychotics in SZ patients. However, the underlying biological mechanism by which tobacco smoke improves symptoms in SZ remains unclear. This study was designed to examine the effects of tobacco smoke on antioxidant enzyme activities and psychiatric symptoms after receiving 12-week risperidone monotherapy.

**Methods:**

Two hundred and fifteen antipsychotic-naïve first-episode (ANFE) patients were recruited and treated with risperidone for 3 months. The severity of the patient’s symptoms was assessed by the Positive and Negative Syndrome Scale (PANSS) at baseline and at post-treatment. Plasma SOD, GSH-Px, and CAT activities were determined at baseline and follow-up.

**Results:**

Relative to nonsmoking patients with ANFE SZ, patients who smoked had higher baseline CAT activity. In addition, among non-smokers with SZ, baseline GSH-Px was associated with clinical symptom improvement, while baseline CAT was associated with positive symptom improvement in smokers with SZ.

**Conclusion:**

Our findings demonstrate that smoking affects the predictive role of baseline SOD, GSH-Px, and CAT activities on clinical symptom improvement in patients with SZ.

## 1 INTRODUCTION

Cigarette smoking in patients with a mental disorder is common. Previous evidence has shown that the prevalence of smoking is around 70-80% in chronic patients with schizophrenia (SZ) and 58% in patients with first-episode psychosis [1, 2], which is higher than the general population [3]. Moreover, patients with SZ who smoke inhale heavier and for longer durations, relative to the general population [4]. They also consume more cigarettes and are more severely dependent on nicotine than smokers without SZ [5]. It was revealed that smoking increases the risk of death from cardiovascular disease in patients with SZ by 86% over twenty years, with a 20-year all-cause mortality risk of 30% [6]. Although the association of tobacco smoke with SZ has been well established, the mechanisms are complex and not yet clear.

The damage caused by smoking is mainly attributed to a high concentration of oxidants to cause cell injury by a variety of mechanisms, such as protein inactivation, peroxidation of lipid membranes, and induction of DNA damage [7]. Additionally, smoking induces a depletion of systemic endogenous antioxidant capacity, resulting in an imbalance between reactive oxygen species and antioxidants, leading to an increased pro-oxidant load and thus also oxidative stress [8]. Protection against these oxidants is provided by free radical scavenger enzymes, such as glutathione peroxidase (GSH-Px), catalase (CAT), and superoxide dismutase (SOD), which are the first line of antioxidant defense system against reactive species-induced damage [9]. Indeed, increased levels or activities of antioxidant enzymes (*e.g*. GSH-Px, CAT, and SOD) have been reported in smokers [10]. Nicotine is the main addictive substance in cigarettes [11, 12]. However, previous studies have also reported that low concentrations of nicotine play a key role in the neuroprotective effect, whereas high concentrations may lead to oxidative stress and neurotoxicity [13]. To date, the role of smoking in activities of SOD, CAT, and GSH-Px enzymes remains unclear, even less clear in SZ, SZ is a complex and progressive brain disease. The brain has high oxygen consumption and an increased rate of oxidative metabolism, contributing to a greater risk for the negative impacts of oxidative stress [14]. A wide body of evidence has been accumulating for the link between oxidative stress, pathophysiology, and symptomatic profile in SZ [15, 16]. Abnormalities in antioxidant enzyme levels and activities have indeed been reported in the blood and brains of SZ patients, after adjusting for those potential confounders [17-20]. Collectively, recent evidence has supported that increased oxidative stress may contribute to chronic, persistent course and poor response to antipsychotics associated with SZ, although it has not been suggested to be the primary cause of SZ.

Even though previous studies have reported a notoriously worsening role of smoking, interestingly, there is emerging evidence that smoking may be a form of self-medication for the clinical symptoms of SZ [21]. Our previous study also found that smoking SZ patients had fewer negative symptoms [22]. Based on the modulation of antioxidant enzyme activity by smoking [23], the close relationship of oxidative stress with the pathophysiology of SZ, and its key role in ameliorating clinical symptoms, we hypothesized that antioxidant enzyme activity at baseline may be a potential prognostic marker in the therapeutic response to antipsychotics in the first episode of drug-free patients with SZ. Furthermore, antioxidant enzyme activity differently predicted symptom improvement in smokers and non-smokers with SZ. Therefore, the aims of the current study were to examine (1) any differences in SOD, GSH-Px, and CAT activities among smokers and non-smokers with SZ; (2) differences in symptom improvement between smokers and non-smokers after 12 weeks of treatment with risperidone; and (3) whether predictive markers such as SOD, GSH-Px and CAT were different in the therapeutic response among smokers and non-smokers.

## 2 MATERIALS AND METHODS

### 2.1 Subjects

Two hundred and fifteen patients with SZ were treated with oral risperidone monotherapy for three months in this study. Patients were diagnosed with SZ on admission according to the Structured Clinical Interview I for DSM-IV (SCID-I) criteria. The inclusion criteria included: first episode; aged between 16 and 45 years; duration of illness < 5 years; and drug-free or cumulative antipsychotic drug exposure < 2 weeks.

One hundred and twenty-five healthy controls were also recruited after screening by SCID interview to exclude potential mental illness. In addition, a complete set of physical examinations and laboratory tests, and medical history were obtained from all subjects to exclude those with major medical comorbidities. The control subject was also excluded if his/her first-degree relative was diagnosed with a psychiatric disorder.

The protocol was approved by the Ethical Committee of Beijing Huilongguan Hospital. All subjects provided written informed consents.

### 2.2 Clinical Measures

All patients were treated with flexible doses of risperidone (4-6 mg/day) for 3 months. Doses of risperidone were determined based on clinical status and psychopathological symptoms. Clinical symptoms were assessed on the Positive and Negative Syndrome Scale (PANSS) by six psychiatrists. After a training course on how to use the PANSS scale, they maintained a correlation coefficient >0.8 to ensure the reliability of the repeated assessments between them on the total score. Then, they assessed the severity of symptoms using this scale [24]. PANSS was evaluated at baseline and at Week 12.

A standardized questionnaire was used to collect smoking data. While patients were in the hospital, nurses took patients who smoked outside at a set time each day to smoke. All patients received the same diet and exercise program.

### 2.3 Antioxidant Enzyme Measurement

Fasting plasma SOD, GSH-Px, and CAT activities were determined by two experienced technicians. SOD activity was measured using the assay of the inhibition of superoxide-induced formation of nitrite from hydroxylamine. *The* GSH-Px activity was measured by adding H_2_O_2_ to the mixture containing glutathione reductase, reduced GSH, and reduced nicotinamide adenine dinucleotidephosphate (NADPH). CAT catalyzes the transformation of hydrogen peroxide to water and oxygen. CAT activity was measured by monitoring the decreased absorbance spectrophotometrically at 240 nm due to the degradation of hydrogen peroxide.

### 2.4 Statistical Analysis

In this longitudinal study, the last-observation-carried-forward method (LOCF) was used to impute values for the missing observations after Week 8. Since most variables were normally distributed (Shapiro-Wilk test), demographic variables and antioxidant activities were compared between smokers and non-smokers by using the *X*^2^ test or analysis of variance (ANOVA) in patients and controls. Repeat measured ANOVA was carried out to compare the clinical symptoms and enzyme activity between smokers and nonsmokers. The associations between antioxidant activities and improvement in clinical symptoms were analyzed among smoking and non-smoking patients. Linear regression analysis was conducted to identify the factors related to symptom improvements in smokers and non-smokers, respectively.

All data were analyzed by using SPSS software (version 22.0), The significance threshold was set at 0.05.

## 3 RESULTS

### 3.1 Baseline Demographic and Antioxidant Enzyme Activities

At baseline, patients had higher SOD and lower GSH-Px activities than healthy controls (*p* < 0.05), but no difference in CAT activity. Smokers had higher age of onset, older age, and greater levels of negative symptoms (*p*_all_ < 0.05) (Table **1**). In addition, smokers had a higher CAT activity than nonsmokers (F = 4.2, *p* = 0.041). No significant correlations between CAT, SOD, and GSH-Px and age, BMI, age of onset, or educational levels were observed in all patients (*p*_all_ > 0.05).

In controls, there were no significant associations between the activities of antioxidant enzymes and smoke (*p*_all_ > 0.05) (Table **1**). Among smoking HCs, there were significant associations between age and GSH-Px activity (r = -0.48, *p* = 0.002), SOD and age (r = -0.33, *p* = 0.03) and BMI (r = -0.42, *p* = 0.008). Among non-smokers in the HC group, there were significant associations between CAT activity and age (r = 0.34, *p* = 0.005) and education years (r = -0.24, *p* = 0.05), as well as between age and GSH-Px activity (r = -0.30, *p* = 0.025) and education years (r = 0.35, *p* = 0.008).

### 3.2 Alterations in SOD, CAT, and GSH-Px Activities and Improvement in Symptoms after Treatment

**Fig. (1):**
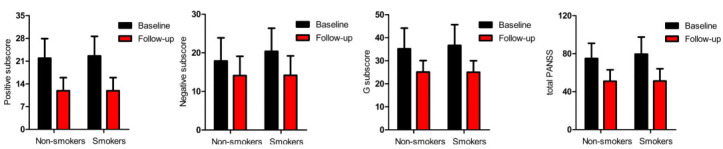
Clinical symptoms were significantly improved after treatment with risperidone in smoking and non-smoking patients with schizophrenia. Smoking patients showed a greater improvement in negative symptoms than nonsmoking patients (*p < 0.05).

Risperidone significantly improved the psychiatric symptoms (*p*_all_ < 0.01). Additionally, smokers had greater improvements in negative symptoms compared to nonsmokers (F = 6.7, *p* = 0.01) (Table **2**), (Fig. **1**).

After 12 weeks of treatment, SOD activity was significantly increased in both smokers and nonsmokers. There were no differences in CAT and GSH-Px activities before and after treatment in both groups (Table **3**). Repeated ANCOVA showed no significant differences in the increases in three antioxidant enzyme activities among smoking and nonsmoking patients (*p*_all_ >0.05).

### 3.3 Relationship of Baseline SOD, CAT, and GSH-Px Activities with Symptom Improvement

At baseline, SOD, GSH-Px and CAT activities did not correlate with average scores on the positive and general psychopathology scales and total scores in smokers and non-smokers with SZ (*p*_all_ > 0.05).

**Fig. (2):**
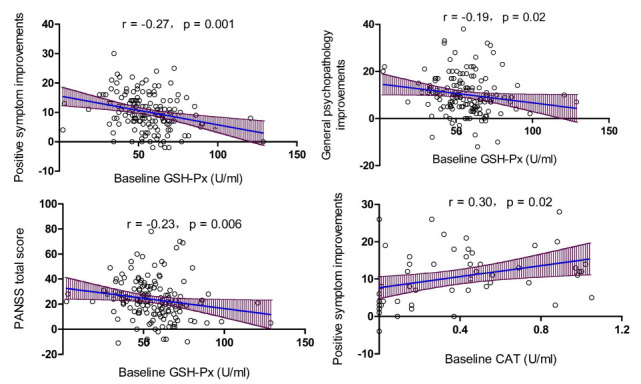
Baseline GSH-Px activity was negatively associated with the improvement in positive symptoms, general psychopathology and PANSS total score in non-smoking patients with schizophrenia (all *p* < 0.05). In addition, baseline CAT activity was positively associated with the improvement in positive symptoms in smoking patients with schizophrenia (*p* < 0.05).

The associations of baseline antioxidant enzyme activities with improvements in clinical symptoms were then analyzed and showed that in nonsmoking patients, baseline GSH-Px activity was negatively correlated with reductions in positive symptoms (r = -0.27, *p* = 0.001), general psychopathology (r = -0.19, *p* = 0.02) and PANSS total score (r = -0.23, *p* = 0.006) (Fig. **2**). While, in smoking patients, baseline CAT activity was positively associated with reductions in positive symptoms (r = 0.30, *p* = 0.02) (Fig. **2**). Further multiple regression analyses confirmed that baseline GSH-Px and CAT activities were a predictor of symptom improvement after controlling for age at onset, age, and baseline BMI. In addition, we reanalyzed the results by a responder status of the patients (50% reduction in the PANSS) and found that the associations remain significant (*p*_all_ <0.05).

Moreover, AUCROC analyzed the AUC value for each antioxidant enzyme. Our results showed that baseline GSH-Px activity had an AUC value of 0.63 for symptom improvement in non-smoking patients (*p* = 0.026, 95% CI = 0.51-0.75), and baseline CAT activity had an AUC value of 0.32 for symptom improvement in smokers (*p* = 0.04, 95% CI = 0.18-0.45).

## 4 DISCUSSION

This is the first cohort study in which the effects of smoking on antioxidant enzyme activities and symptom improvements were assessed in patients with SZ following 12-week risperidone treatment. Our study showed that 1) at baseline, smokers with SZ had significantly higher CAT activity than nonsmokers; 2) compared to non-smokers, smokers improved more negative symptoms after treatment with risperidone for 12 weeks; and 3) after treatment, baseline GSH-Px was associated with symptom improvements in nonsmokers, while in smokers, the baseline CAT was associated with the reductions of positive symptoms.

We found that at the onset of SZ, negative symptoms were more severe in smokers than in non-smokers with SZ, which supports a close relationship between smoking and severe clinical symptoms in SZ. However, we found greater improvements in negative symptoms in smokers relative to nonsmokers after treatment, but not in positive symptoms or general psychopathology. Although there were significant differences in age, education years, and onset age, when all were added as covariates, differences in improvement in negative symptoms remained significant. Our findings were consistent with earlier studies [25-28]. It is hypothesized that smoking could increase the levels of dopamine, which would then alleviate the symptoms of patients [29-31]. Animal studies also support that systemic administration of nicotine can increase the activity of the dopaminergic system and reduces dopamine degradation [32]. Furthermore, a possible role of smoking on risperidone pharmacokinetics and metabolism is speculated. It is well-known that smoking induces the activity of CYP1A2 leading to decreased plasma levels of risperidone in a dose-dependent manner with the number of cigarettes smoked each day [33, 34]. Besides, higher clearance rates of risperidone have also been reported in smoking individuals by inducing CYP1A2 activity [35, 36]. Several studies have reported that smokers received higher doses of risperidone compared with non-smokers [36]. Contrary to our findings, a study in chronic patients with SZ reported no significant difference in negative symptoms between smokers and non-smokers, but the greater number of cigarettes smoked, the fewer the negative symptoms [22]. One possible reason to explain this discrepancy between the two studies is that the participants recruited in these studies (first episode drug-naïve *vs.* chronic) and the study type (cohort study *vs.* case-control study) were different.

We found that CAT activity at baseline was different in smokers than in non-smokers, suggesting that tobacco smoke showed an impact on the antioxidant enzyme activities in the onset stage of SZ. CAT is one of the major enzymes in the enzymic antioxidants *in vivo*. We speculated that in smokers, the excess free radicals produced by tobacco smoke or the pathophysiology of SZ induces an increase in CAT activity as a compensatory mechanism. Smoke is related to significant systemic oxidative stress, leading to an increase in antioxidant enzyme activity in peripheral blood [37]. Indeed, smoking has been shown to modulate CAT activity in earlier studies [38-40]. In conclusion, our findings support that an elaborately regulated redox system in favor of antioxidants exists in the early stage of illness in smokers with SZ.

Notably, we further found baseline CAT can predict improvement in positive symptoms only in smokers with SZ. Namely, the higher the CAT activity at baseline, the greater improvements in positive symptoms of SZ. A complex interaction of smoke, antipsychotic medication, and the pathophysiology of SZ releases more damaging free radicals. CAT can scavenge hydrogen peroxide or organic hydroperoxide in neurons [41]. Thus, patients with higher baseline CAT activity may have a better function in the regulations of antioxidant enzymes and be more likely to recover from the disease after treatment with risperidone. On the other hand, nicotine induces cytochrome P450 enzyme activity and increases the metabolism of antipsychotics. In smoking patients, it is speculated that the relationship between symptom improvement with CAT activity was more complex than in nonsmokers. That is why we found only an association between baseline CAT and therapeutic response in smokers, but not in non-smokers. However, contrary to our expectation, CAT was only related to positive symptom improvement, rather than negative symptoms which were reported to be correlated with tobacco smoke in this study. We speculate that one possible reason for the lack of a relationship between negative symptom improvements and CAT at baseline among smokers and non-smokers was the low statistical power due to the small sample size of smokers and short durations of antipsychotic medications in the present cohort study. It is also possible that we measured the activities of these antioxidant enzymes in peripheral blood and not in the central nervous system.

On the other hand, we found that in nonsmokers with SZ, the baseline GSH-Px activity was negatively associated with improvements in clinical symptoms. We speculated that the higher baseline GSH-Px activity in nonsmokers indicates a more severe abnormality in the redox system due to the pathophysiology of SZ at the early stage of this disease. Patients with lower GSH-Px activity at baseline are likely to have less damage to the neurological function of the brain by the disease. Therefore, those patients’ symptoms were much improved after treatment. Interestingly, our study was consistent with a recent study by Chen *et al.* which reported that altered GSH-Px activity was associated with improvement in clinical symptoms in nonsmokers following antipsychotic treatment [42].

Some limitations should be addressed in this study. First, although antioxidants might be of some clinical relevance, a focus on antioxidants alone appears too narrow in scope. In addition, there were no significant associations between antioxidant enzyme activities and smoking, possibly pointing to the minor role of cigarettes. Second, there was no recognition in this study of the assessment of other important domains such as extrapyramidal as well as metabolic adverse effects, cognition, and subjective perspectives (*e.g*. QOL). The PANSS is merely one of all symptoms of SZ. Third, smoking data were imperfect, which should have included the duration of smoking and the amount of smoking per day (*e.g*. pack years). Fourth, antioxidants are just one of the potential contributors to the pathophysiology of SZ, which remains totally elusive to date. In addition to antioxidant enzymes, there are other antioxidants, such as vitamin E, Moreover, antioxidant levels may be influenced by various factors other than smoking (*e.g*. diet habits, amount of exercise, and BMI). Unfortunately, we did not collect all of these factors in this study, which should be remedied in future studies. In addition, the results of this study were merely correlational, not causal, because the association did not equate to causation. Fifth, it is worthy of mentioning that most critically the standard deviations *versus* the means of antioxidants are large, especially for CAT, indicating substantial inter-individual variability and making them unreliable as unequivocal biomarkers.

## CONCLUSION

In summary, we found that at the onset of the disease, smokers with SZ had significantly higher CAT activity than nonsmokers. We did not find associations between baseline SOD, CAT, and GSH-Px activities and negative symptom improvements in smokers with SZ, but we found a predictive role of CAT for positive symptom improvements. On the contrary, for non-smokers with SZ, GSH-Px at baseline correlated with a positive symptom, general psychopathology, and total score. The differences in the associations between the symptom improvements and enzymes at baseline in smokers and nonsmokers suggest that tobacco smoke at the onset of the disorder may regulate the antioxidant enzyme system in SZ and impact the therapeutic response to risperidone monotherapy.

## Figures and Tables

**Table 1:** Demographic characteristics and antioxidant enzyme activities in antipsychotics-naive first episode (ANFE) patients with schizophrenia (SZ) and healthy controls (HCs).

**Variable**	**SZ Patients**			**HC Subjects**		
	**Nonsmokers ** **(n = 153)**	**Smokers ** **(n = 62)**	***p* value**	**Nonsmokers ** **(n = 80)**	**Smokers ** **(n = 45)**	***p* value**
Sex (M/F)	66/87	54/8	34.6(<0.001)	37/43	40/5	22.1(<0.001)
Age (years)	26.8 ± 9.4	30.6 ± 8.7	7.1(0.008)	26.5 ± 7.4	29.6 ± 8.6	4.7(0.03)
Education (years)	9.6 ± 3.8	8.6 ± 3.9	3.4(0.07)	10.9 ± 3.0	9.3 ± 3.0	7.5(0.007)
BMI (kg/m^2^)	21.3 ± 3.6	22.0± 3.1	2.0(0.16)	23.2 ± 3.5	24.0 ± 4.9	1.0(0.32)
AOO (years)	25.5 ± 9.3	28.3 ± 9.0	4.0(0.046)	-	-	-
**Antioxidant Enzymes**						
GSH-Px (U/ml)	55.6 ± 17.4	56.5 ± 18.5	0.1(0.74)	64.6 ± 15.8	61.3 ± 17.2	0.9(0.33)
CAT (U/ml)	0.24 ± 0.3	0.34 ± 0.3	4.2(0.04)	0.30 ± 0.43	0.30 ± 0.38	0.2(0.67)
SOD (U/ml)	75.9 ± 9.0	74.0 ± 10.2	1.9(0.17)	63.5 ± 13.7	67.8 ± 11.3	2.9(0.09)

**Abbreviation: ***BMI* Body mass index; *AOO* age of onset.

**Table 2:** Symptom improvements between smokers and nonsmokers on risperidone.

-	**Nonsmokers**	**Smokers**	***p* Value**
**Symptom Improvements**			
P score	10.0 ± 6.3	10.7 ± 7.3	0.5(0.48)
N score	3.8 ± 5.5	6.2 ± 7.5	6.7(0.01)
G score	10.2 ± 9.6	11.7 ± 9.3	1.2(0.28)
Total score	23.8 ± 17.9	28.3 ± 20.2	2.6(0.11)

**Table 3:** Comparisons of clinical symptoms and antioxidant enzymes before and after 12 weeks of risperidone monotherapy.

**-**	**Baseline**		**12-week Follow-up**		**Effect^a^**		
	**Non-smokers**	**Smokers**	**Non-smokers**	**Smokers**	**Time F(p)**	**Smoke F(p)**	**Interaction F(p)**
**Clinical Symptoms**							
*P* score	21.9 ± 6.3	22.6 ± 6.9	11.9 ± 4.7	11.9 ± 4.6	24.9(<0.001)	0.1(0.76)	0.1(0.75)
N score	17.9 ± 6.6	20.4 ± 7.6	14.1 ± 5.5	14.2 ± 6.1	22.2(<0.001)	3.1(0.08)	7.6(0.006)
G score	35.2 ± 9.6	36.7 ± 10.3	25.1 ± 5.9	25.0 ± 6.6	15.3(<0.001)	0.3(0.57)	0.7(0.40)
Total score	74.9 ± 16.6	79.5 ± 19.5	51.0 ± 12.9	51.2 ± 13.7	28.4(<0.001)	1.6(0.21)	2.0(0.16)
**Antioxidant Enzymes**							
SOD (U/ml)	75.8 ± 9.1	74.0 ± 10.2	77.0 ± 7.4	75.0 ± 10.8	10.8(0.001)	2.3(0.13)	0.2(0.66)
GSH-Px (U/ml)	55.4 ± 17.3	56.5 ± 18.5	47.9 ± 14.1	52.6 ± 15.2	3.2(0.08)	1.5(0.23)	1.7(0.20)
CAT (U/ml)	0.24 ± 0.27	0.34 ± 0.34	0.30 ± 0.31	0.38 ± 0.28	0.09(0.34)	4.6(0.03)	0.14(0.71)

## Data Availability

Not applicable.

## LIST OF ABBREVIATIONS

ANFE: Antipsychotic-naïve first-episode
CAT: Catalase
PANSS: Positive and Negative Syndrome Scale
SOD: Superoxide Dismutase
SZ: Schizophrenia
